# Overwintering survivorship and growth of young-of-the-year black sea bass *Centropristis striata*

**DOI:** 10.1371/journal.pone.0236705

**Published:** 2020-08-24

**Authors:** Adam F. Younes, Robert M. Cerrato, Janet A. Nye

**Affiliations:** 1 School of Marine and Atmospheric Sciences, Stony Brook University, Stony Brook, NY, United States of America; 2 Institute of Marine Sciences, University of North Carolina at Chapel Hill, Morehead City, NC, United States of America; Department of Agriculture, Water and the Environment, AUSTRALIA

## Abstract

Overwintering conditions have long been known to affect fish survival and year-class strength as well as determine the poleward range limit of many temperate fishes. Despite this known importance, mechanisms controlling overwintering mortality are poorly understood and the tolerance of marine fishes to the combined effects of winter temperature, salinity, and size is rarely quantified. In recent years, higher abundances of the temperate Serranid, black sea bass *Centropristis striata*, have been observed at latitudes further north than their traditional range suggesting that warming water temperatures, particularly during winter, may be facilitating the establishment of a population at more northern latitudes. To examine overwintering survival of *C*. *striata*, the combined effects of temperature, salinity and body mass were quantified in laboratory experiments. We identified 6°C as the lower incipient lethal temperature for *C*. *striata*, below which fish cease feeding, lose weight rapidly and die within 32 days. A short cold exposure experiment indicated that temperatures below 5°C resulted in mortality events that continued even as the temperature increased slowly to 10°C, indicating that even short cold snaps can impact survival and recruitment in this species. Importantly, fish in lower salinity lived significantly longer than fish at higher salinity at both 3°C and 5°C, suggesting that osmoregulatory stress plays a role in overwintering mortality in this species. Size was not a critical factor in determining overwintering survival of young-of-the-year (YOY) *C*. *striata*. Overwintering survival of YOY *C*. *striata* can be effectively predicted as a function of temperature and salinity and their interaction with an accelerated failure model to project future range limits. Identifying temperature thresholds may be a tractable way to incorporate environmental factors into population models and stock assessment models in fishes.

## Introduction

Temperature has long been known as a controlling, directive, and lethal factor in marine fishes [1, 2]. This means that within a species’ lethal temperature range, temperature controls its metabolic scope for growth and can also be a cue for critical behaviors like spawning and migration. Other factors like dissolved oxygen, salinity, and pH also influence growth and survival. In particular, salinity is known to be a masking factor because it changes the way in which energy is used by the fish, can reduce metabolic scope and shift the optimal temperature for growth and performance [1, 2]. Yet, environmental tolerances are poorly quantified for most marine fishes especially in ways that are adequate to predict spatial distribution and demographic processes critical to population assessment. Most studies, particular those focused on aquaculture applications, often seek to identify the optimal environmental conditions for growth, but a wealth of evidence suggests that it is the extreme temperatures and the combined effect of multiple stressors that determine biogeography and population dynamics of ectotherms [3–5].

Overwintering is an important source of mortality for many temperate marine fishes, but the mechanisms underlying mortality at low temperatures are not well-understood. There are three potential mechanisms for temperature-induced death: 1) a disturbance to the central nervous system, 2) osmoregulatory failure and 3) starvation [2]. These mechanisms are not necessarily mutually exclusive at low temperatures, but the time to death as a function of temperature is an important clue as to the mechanism of overwintering mortality. Rapid death typically indicates the first mechanism, damage to the central nervous system, which seems to be the most sensitive tissue at both high and low temperatures [2, 6]. At the other extreme, delayed death after weeks to months of cold exposure, is likely related to starvation and potentially related to size and energy stores. The role of osmoregulatory failure in overwintering mortality occurs at some intermediate exposure time, but our understanding is hindered by few experiments that quantify the combined effect of temperature and salinity on mortality rates.

Osmoregulation is critical to maintaining homeostasis in fishes as they are rarely in water that is isosmotic with their bodies. Marine fish must constantly drink water and excrete salts in order to maintain homeostasis. The ability of fishes to maintain homeostasis is reliant upon active ion pumping, a temperature dependent process, and passive ion diffusion, a temperature independent process [7]. When temperatures approach a fishes’ lower thermal tolerance active ion pumping is hindered and homeostasis cannot be maintained resulting in ion concentrations in the blood plasma similar to the surrounding water that eventually causes death [8, 9]. If osmoregulatory failure is a mechanism of overwintering mortality, we hypothesize that mortality rates should be highly dependent on not just temperature, but salinity and that mortality occurs at a rate greater than starvation mortality, but not as quickly as central nervous system failure.

Previous work on overwintering mortality in fishes has focused primarily on the first year of life [10–13]. For many species in seasonal environments, winter months are associated with little or no growth, weight loss and size-selective mortality [14–16]. Consequently, recruitment and year-class strength can be strongly controlled by environmental conditions in the winter months [11, 17–19]. Furthermore, species assemblages respond rapidly to winter temperature and as such, it is likely important for explaining biogeography and range shifts in the context of global climate change [20–22]. Warming winter water temperatures in response to anthropogenic climate change may enable the poleward range expansion of many temperate species. The Northeast United States (US) is an ideal place to understand the role of winter temperature in species distribution and abundance in the context of climate change as this region experiences some of the widest seasonal variations in water temperature and recent rapid warming in this region has already affected the ecosystem [23–25].

The Northwest Atlantic is one of the fastest warming regions in the ocean [23]. *Centropristis striata*, the most northerly-distributed grouper species (Family Serranidae) in the Northwest Atlantic [26], has seemingly responded to the warming in this region as evidenced by higher abundances in the northern extent of their historic range [27] and by expanding their geographic range to the extent that they are now commonly found in the southern Gulf of Maine during spring and summer [28–30] where they had only been found occasionally in the past [26]. One potential mechanism for this range expansion is that overwintering mortality is reduced as temperatures on the Northeast shelf have increased. With warmer waters not only might mortality be reduced, but there may be an added benefit of warmer winters if the need to migrate is reduced. As warming continues, *C*. *striata* may continue to establish their center of biomass further north, providing a new fishery in regions where one did not exist previously, potentially with substantial ecological consequences and requiring adaptive management practices in a potentially emerging fishery.

*C*. *striata* supports recreational and commercial fisheries throughout its range from the northern end of the Florida peninsula to Massachusetts. The coastwide population is thought to consist of two stocks divided at Cape Hatteras, North Carolina [31, 32]. The stock north of Cape Hatteras is genetically distinct from the stock south of Cape Hatteras [33] so they are assessed and managed separately. In the northern stock, seasonal temperature variability elicits an offshore and southward migration that is not observed in the southern stock presumably as a behavioral mechanism to avoid suboptimal or lethal winter temperatures [34, 35]. Tagging studies show that adult C. *striata* begin moving offshore in late summer, continue leaving nearshore waters fairly steadily throughout the fall and that fall mixing events likely cue many adult *C*. *striata* to move offshore [36, 37].

In the spring, adult *C*. *striata* move inshore and remain in coastal areas and estuaries throughout the summer. Spawning occurs in the late summer and early fall. Young of the year (YOY) *C*. *striata* can be found in bays and estuaries in the late summer to early fall and move out of estuaries in the fall. It is thought that, like the adults in the northern subpopulation, YOY *C*. *striata* also move offshore and south in response to temperature; however, little is known about their distribution once they leave estuaries. The ability of YOY *C*. *striata* at the northern extent of their range to survive their first winter depends on their temperature and salinity tolerance, the magnitude and duration of winter, as well as the timing and extent of their seasonal migration offshore to escape colder, lower salinity estuarine and nearshore waters [38].

Even though the combined effects of temperature, salinity and size among other factors impact growth and survivorship, they are often considered in isolation and this hinders both our understanding of mechanisms of overwintering mortality as well as our ability to quantify the population-level and ecological consequences of this important process (6). Previous work has attempted to identify temperature optima and thresholds for different stages of *C*. *striata*, but has resulted in conflicting results likely due to different methodology and/or lack of consideration for multiple environmental factors [14, 39–43].

Statistically robust and mechanistically-based models are needed to better understand the relative importance of different factors influencing overwintering mortality and this need has only become more important as society considers potential range expansions of this and other species in response to anthropogenic climate change. Our goal was to identify mechanisms of winter mortality in *C*. *striata* and develop a model of overwintering mortality as a function of time, temperature, salinity and size with a statistical approach that can ultimately be applied to other fishes. In this study, YOY *C*. *striata* were exposed to various temperatures and salinities in a controlled laboratory setting to develop an accelerated failure time model quantifying the rate of mortality as a function of temperature, salinity, and size. Growth rates were also measured during the overwintering experiments. In addition, a short cold exposure experiment was conducted to identify the temperatures on which to focus and to elucidate mechanisms of overwintering mortality.

## Materials and methods

### Collection and animal husbandry

All work was approved under Stony Brook University Institutional Animal Care and Use Committee (IACUC) protocol 2015-2161-NF-FI-2.16.18 and all researchers handling fish were trained in animal care and cleared to be study personnel under this protocol number. YOY *C*. *striata* were collected from the coastal Atlantic Ocean off of Oak Beach, New York US (40^o^ 38’ 21.7” N, 73 ^o^ 18’ 28.7” W) by seine and from the Peconic Estuary by bottom trawl in Long Island, New York USA between August and October 2013 and 2014 by A. Younes under New York Department of Environmental Conservation Scientific collecting permit #1409. Fish from Peconic Estuary were captured during the New York State Department of Environmental Conservation’s bottom trawl survey (see S1 File). Fish were transferred to Flax Pond Laboratory in East Setauket, NY where they were held near the temperature and salinity at which they were captured, 20°C and a salinity of 29 PSU, in four 1,100-liter recirculating holding tanks prior to the start of the experiment.

To acclimate fish in all experiments, salinity was first changed by no more than 1 PSU per day and then temperature was reduced by not more than 1°C per day until the experimental temperature and salinity were reached. The start dates of experiments were staggered so that fish were acclimated at the same rate. When the desired experimental temperature was reached, fish were randomly selected from the holding tanks, weighed, total length (*L*_T_) measured and placed into individual containers. Photoperiod was kept at 9 h light—15 h dark to simulate winter. During the experiment, fish were held in individual 4-L containers each with its own individual water flow providing 1-L min^-1^ flow to each container. All containers were submerged in a 650-L sea table serving as a water bath to maintain temperature. Each temperature and salinity treatment combination had its own sea table cooled by an in-line chiller. Fish were fed finfish starter pellets *ad libitum* once daily and any uneaten food and feces were siphoned once daily. Each fish was provided shelter in the form of a small PVC pipe (8.90 cm height x 3.80 cm circumference). YOY *C*. *striata* were checked once per day. If fish had completely lost equilibrium they were observed for approximately one hour and if they could not equilibrate they were sacrificed with an overdose of MS-222. However, most fish were found already deceased. Fish were measured for growth either at the end of experiment if still alive or post-mortality. Temperature and salinity were monitored daily (Table 1). Water changes were done at least once per week.

**Table 1:** Mean and standard deviation (Mean ± SD) of temperature, salinity and initial mass during the overwintering experiments of YOY *C*. *striata*.

Treatment	n	Duration of Experiment (days)	Temperature (°C)	Salinity (PSU)	Initial Mass (g)
3°C, 15	36	7	3.9 ± 1.20*	15.0± 0.11	3.39 ± 1.02
3°C, 35	36	3	3.2 ± 0.42	34.9 ± 0.29	4.02 ± 1.50
5°C, 15	36	32	5.2 ± 0.47	1489 ± 0.29	4.23 ± 2.11
5°C, 35	36	22	4.7 ± 0.26	34.7 ± 0.24	4.18 ± 2.08
6°C, 29	26	80	6.3 ± 1.22	29.1 ± 0.16	5.79 ± 2.20
6°C, 34	26	80	6.1 ± 1.29	34.1 ± 0.19	5.57 ± 1.99
8°C, 29	26	75	8.3 ± 0.54	29.1 ± 0.12	6.42 ± 2.18
8°C, 34	26	75	8.4 ± 0.64	34.1 ± 0.18	5.25 ± 2.47
10°C, 29	26	41	10.3 ± 1.35	29.2 ± 0.16	7.14 ± 2.74
10°C, 34	26	87	9.9 ± 1.79	33.9 ± 0.57	5.88 ± 3.39
12°C, 29	26	84	12.3 ± 1.00	29.3 ± 1.09	6.11 ± 2.14
12°C, 34	26	84	12.1 ± 0.26	34.2 ± 0.19	5.92 ± 1.79

* Indicates the treatment with temperature spike after 3 days.

### Growth and survival experiments

Two sets of experiments were conducted at two different times, all to quantify overwintering mortality as a function of temperature, salinity and size. The temperature levels for the first experiment were 6, 8, 10, and 12°C and the salinity levels were 29 and 34psu. The following year a second set of fish were collected in the same manner and additional experiments were conducted at temperatures of 3 and 5°C and salinities of 15 and 35 psu. Collection and animal husbandry was the same for all experiments except that the fish kept at the 3, 5 and 6°C were held in a controlled-temperature room using air to cool the water baths rather than in sea tables with inline chillers as described above. Growth and survivorship was estimated for all, but the 10°C treatments. Weights from individual fish taken before and after the experiment were used to calculate specific growth rate (SGR, % mass day⁻^1^) in each treatment where SGR = (ln final mass–ln initial mass) x 100/t and t is the number of days survived.

In the first set of experiments, little to no mortality occurred after 40 days so we conducted an experiment on the 10°C and 29 PSU treatment. To better identify the lower lethal temperature and inform the next set of experiments, the water temperature was reduced by no more than 1°C day^-1^ over an 11-day period until a temperature of 3.5°C was reached and the first death occurred. The fish were held at this temperature for 9 days. The temperature was then increased by ~1°C day^-1^ back to 10°C to test the hypothesis that the lower lethal limit had been reached and that cold temperatures had caused irreparable tissue damage. If fish continued to die even as temperature increased, this was an indication that irreparable tissue damage occurred.

### Statistical analysis

Since experiments occurred in two phases, growth during the two phases of experiments were analyzed with Analysis of Variance (ANOVA) separately. Differences in SGR were analyzed using a two-way ANOVA with temperature and salinity as factors. Significant differences among treatment combinations were examined using Tukey’s multiple comparison test (α = 0.05).

To develop a mechanistic model of overwintering mortality in this species, we used accelerated failure models with censoring, a parametric survival method that is used frequently in medical research and engineering to describe processes that accelerate or decelerate with longer exposure to a stressor and have been used with other marine organisms [44, 45]. An advantage of survival analysis is that it allows the user to pick distributions that do not have constant variance or normal errors since time until death usually changes the longer the subject is exposed to the treatment [46]. If a replicate does not die or the time of a mortality event is unknown, it is considered censored. In our experiments, censored fish are those that did not die during the experiment.

In the models, the predictor variables of mass, salinity and temperature were treated as continuous variables and we tested the fit of models with all possible combinations of these variables and their two-way interactions [47]. Models were fit to eight different parametric distributions (Weibull, log-logistic, lognormal, logistic, extreme, Gaussian, exponential and Rayleigh) to obtain the best-fitting model. The first six of these distributions estimate a scale parameter to determine the shape of the curve and as such an extra parameter must be estimated. The models assuming the exponential and Rayleigh distributions fix scale at 1 and 0.5 respectively so have one less parameter to estimate. The best model was selected using the Akaike’s information criterion (AIC) following the approach outlined in Burnham and Anderson [48]. AIC uses the maximum likelihood and penalizes for the number of parameters to determine the most likely model using the following equation:

$$AIC_{i}=‐2logL_{i}+2k$$

where *L*_*i*_ is the maximum likelihood for model *i* and *k* is the number of parameters. Model selection was aided by calculating *ΔAIC* which is the difference between *AIC*_*i*_ and the model with the lowest AIC. Akaike weights (*wAIC*_*i*_) gives the probability that each individual model is best given the data and set of models being considered and was used for model selection using the following equation:

$${wAIC}_{i}=\frac{e[-0.5(\Delta_{i}AIC)]}{\sum_{i=1}^{n}e[-0.5(\Delta_{i}AIC)]}$$

where $\Sigma_{i=1}^{n}{wAIC}_{i}=1$. The best performing model was verified by comparing observed versus predicted mortality to visualize how well the model fit to observed data.

Almost all the fish survived in the experiments where temperatures were greater than 5°C so survival analysis was conducted using only the 3° and 5°C experiments to quantify mortality at temperature and salinity using the Survival library in R [49]. The Kaplan Meier estimator approximates the survival function using only the observations and without developing a full accelerated failure model [50]. We used it to test for differences among treatments along with a Wilcoxon test [51] using the R function *survdiff* (α = 0.05). Data and code can be found at 10.6084/m9.figshare.12011832.

## Results

### Growth

**Fig 1:**
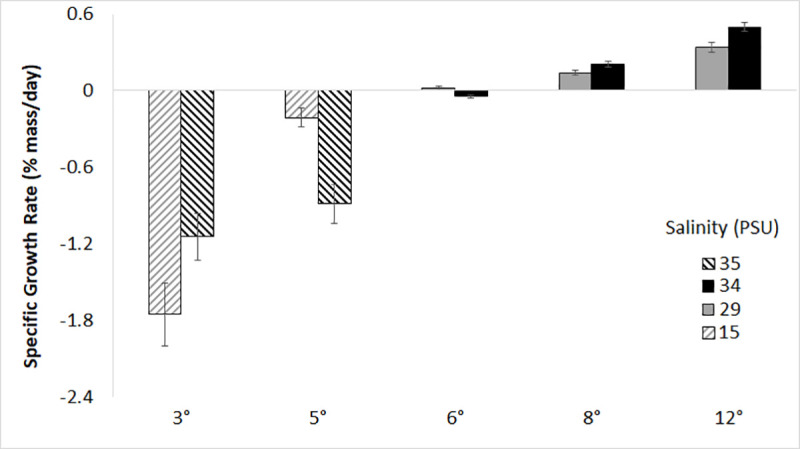
Specific growth rates (%mass day^-1^) of YOY *C*. *striata* at five temperatures and four salinity treatments. The 12°, 8° and 6°C treatments were conducted at different times from the 3° and 5°C treatments so are analyzed separately. Error bars are ± 1 SD.

Growth was positive at 8°C and 12°C, was close to zero at 6°C, and weight loss occurred at 3°C and 5°C (Fig 1). In the first set of experiments, there was a significant interaction between temperature and salinity on growth likely because growth at 34 PSU was higher than at 29 PSU at 8 and 12°C, but lower at 6°C (F_2,144_ = 9.737, p < 0.001). Based on Tukey multiple comparisons, growth of YOY *C*. *striata* was significantly higher for the 12°C treatments compared to 8°C and 6°C treatments (p < 0.001) and growth at 8°C was higher than growth at 6°C. Growth at 12°C was significantly lower at 29 PSU compared to 34 PSU (p < 0.001), but at 8°C and 6°C there was no statistical difference in growth across salinity treatments (p > 0.05). Weight loss (SGR = -0.042 ± 0.056% mass day^-1^, mean ± SD) was observed for the 6°C and 34 PSU treatment. Fish at all temperatures between 6–12°C were observed consistently feeding during the course of the experiment.

In the second set of experiments at 3°C and 5°C, fish were never observed feeding even though food was offered daily, growth was negative at both temperatures, and the interaction between temperature and salinity was again significant (F_1,73_ = 7.955, p < 0.01). According to Tukey multiple comparisons, weight loss at 5°C and 15 PSU was significantly less than all other treatments (p < 0.01, Fig 1). No other pairwise comparison was statistically significant.

### Survivorship

**Fig 2:**
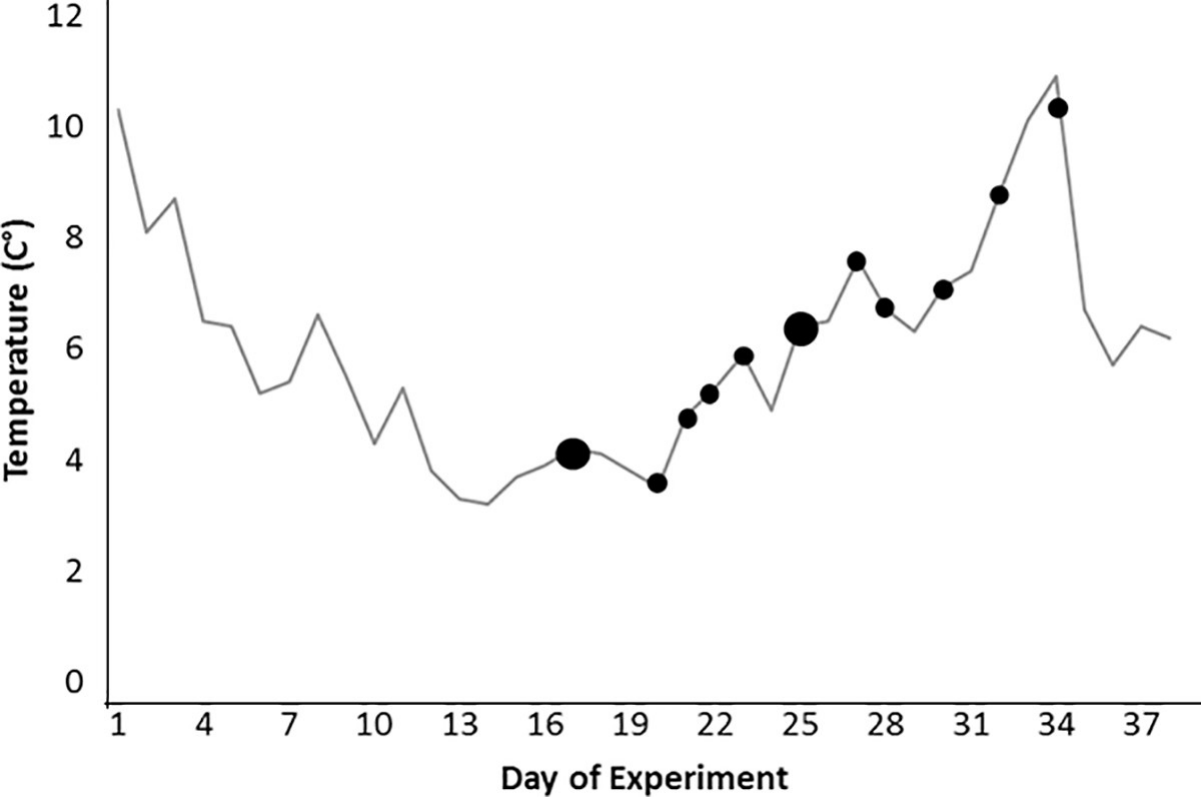
Temperature (gray line) and days with a single fish mortality (black circles) during the low thermal exposure experiment of YOY *C*. *striata*. Large circles indicate two deaths.

We expected at least some mortality in the first set of experiments, but only a few fish died in the 6, 8, 10, and 12°C treatments. Rather than measure growth in the 10°C experiment we lowered temperature to just below 4°C in order to approximate the lower lethal limit (Fig 2). No fish died in the first eleven days as temperature was reduced, but once temperature dropped below 4°C, the first mortality event occurred on the 16^th^ day of the experiment. Three fish (11.5%) suffered mortality after nine days of exposure to temperatures in the range of 3–4°C. After these mortality events occurred the temperature was gradually increased back up to 10°C, but an additional 10 fish (38.5%) died even as the temperature increased (Fig 2). Half of the fish (13 of the 26) died within just nine days below 4°C prompting us to do a second set of experiments at 3 and 5°C.

**Fig 3:**
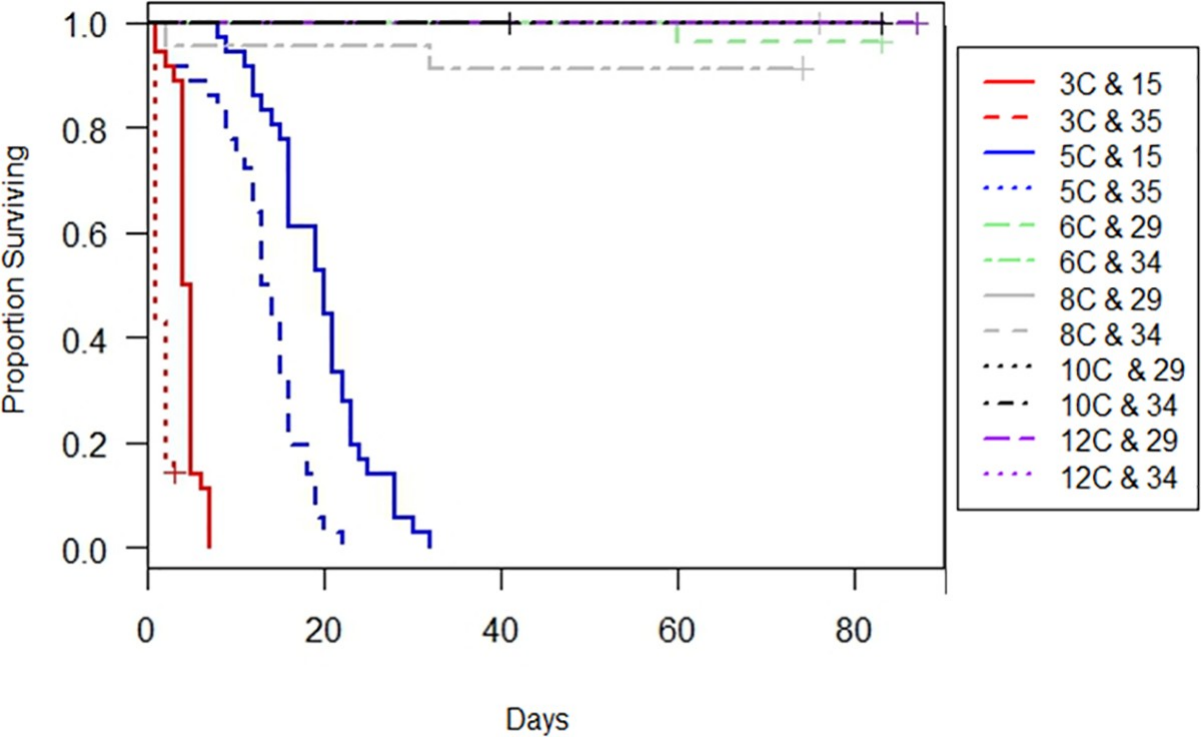
Kaplan-Meier curves depicting proportion of YOY *C*. *striata* surviving in each treatment over the duration of the experiment. Crosses indicate the end date of experiments where some data were censored for survivorship models. For all treatments above 5°C the lines overlap as very little mortality occurred.

In the second set of experiments all fish died within 32 days (Fig 3). For the 3°C and 5°C experiment, the Wilcoxon test indicated that survivorship among treatments was significantly different (Wilcoxon, *X*^2^ = 211, df = 3, p < 0.001). Higher survivorship was observed in the low salinity treatments at both 3°C and 5°C compared to the high salinity treatments (Fig 3). Survival at 5°C and 15 PSU was 100% for seven days and decreased steadily for the remainder of the experiment. Mortality in the 5°C and 35 PSU treatment was more abrupt compared to the lower salinity treatment at the same temperature. Median survivorship was 19 and 13 days for the 5°C treatments at low and high salinity, respectively. Fish in the 3°C and 15 PSU treatment had median survivorship of 4.5 days. Fish exposed to 3°C and 35 PSU suffered acute mortality, surviving a maximum of 3 days, but most dying within one day. Fish exposed to 5°C survived a maximum of 32 days and 22 days at salinities of 15 and 35 PSU, respectively.

Temperature and salinity were relatively constant throughout the 3°C and 5°C experiment (Table 1); however, a cold room malfunction in the 3°C and 15 PSU treatment caused the temperature to spike to 17°C between days two and three. Since 85% (n = 30) of the fish were dead on day three the remaining fish (n = 6) were treated as censored observations in the survival analysis. All 6 fish eventually died within 13 days even though the temperature increased slightly and then decreased again to 3°C. The temperature increased slightly to 6.2°C in the 3°C and 35 PSU experiment on day 3, but was brought back down on day 4 and all fish died by Day 7 so we did not censor any observations.

**Fig 4:**
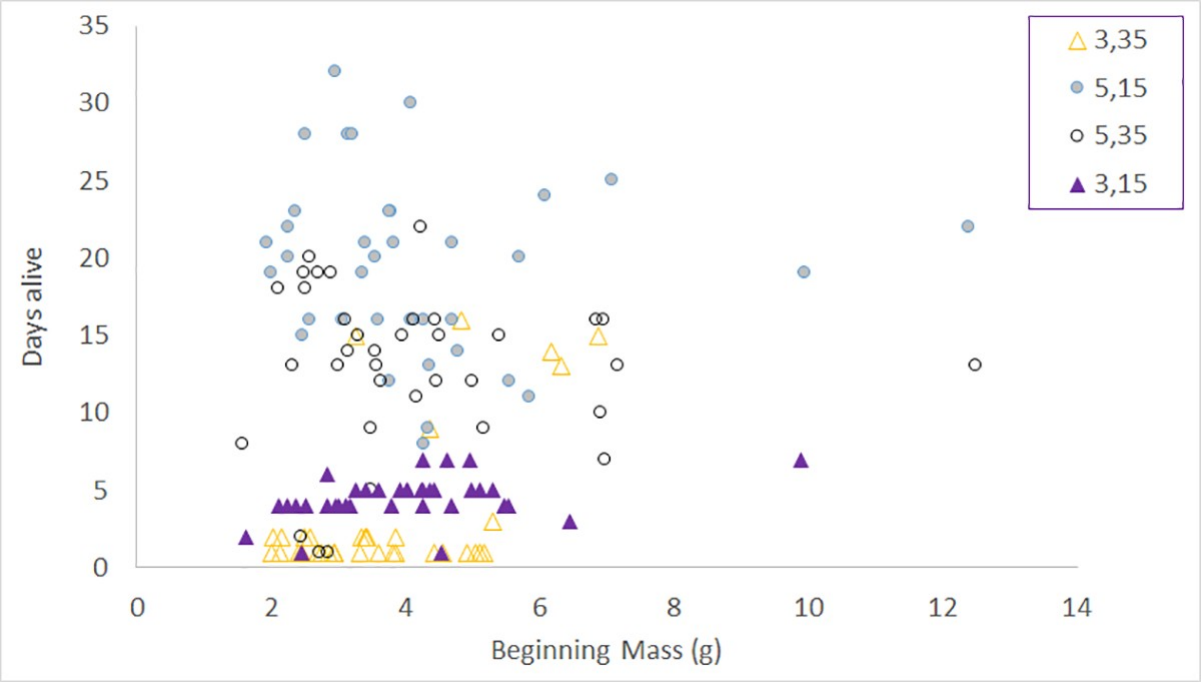
Days until mortality as a function of size for the 3°C and 5°C experiments where 100% mortality occurred.

There was no obvious pattern in mortality as a function of size (Fig 4). However, in the 3°C, 35 PSU experiment all of the fish greater than 6g survived longest (at least 11 days), but all fish <3g died within 3 days (Fig 4).

The Weibull distribution consistently fit the data best for each possible combination of factors (mass, temperature, salinity and their interactions; Table 2). The best models all included temperature, salinity and their interaction. For each distribution when the interaction of salinity and temperature was taken out of the model the AIC increased considerably (Table 2). The worst models were those that only incorporated mass and/or salinity and not temperature. The four most plausible models based on Akaike weights (*wAIC*) differed only in the way mass was included in the model. The most parsimonious of these excluded mass completely, and only included temperature, salinity, and their interaction. This simple model was second only to a model with temperature, salinity, mass, and all two-way interactions. The top two models were more than twice as probable as the next best two models that also included a temperature x salinity interaction. The model excluding mass was chosen as the best model even though its AIC was slightly higher because Burnham and Anderson [48] recommend only choosing a model with more parameters if the difference in AIC is greater than 2 units. Both models described survivorship similarly, but the more parsimonious model does not require any assumption about mass to apply it (Table 2).

**Table 2:** Number of parameters (k) and AIC values for possible accelerated failure models in the 3°C and 5°C experiment.

			**Distribution**									
	k	Weibull	ΔAIC	*w*_*i*_AIC	Log logistic	Log-normal	Logistic	Extreme	Gaussian	k	Rayleigh	Exponential
Mass * Salinity *Temp (only 2 way interactions)	8	641.0	0	0.396	666.2	708.4	769.1	784.2	788.7	7	677.7	814.8
Temp*Salinity	5	641.4	0.4	0.324	662.3	704.5	764.6	784.4	783.5	4	673.6	809.1
Temp*Salinity*Mass	9	643.0	2	0.146	666.3	708.5	771.0	786.0	790.3	8	679.5	816.6
Temp*Salinity + Mass	6	643.2	2.2	0.132	664.3	705.1	766.6	784.5	785.5	5	675.5	811.1
Temp*Mass + Salinity	6	671.5	30.5	<0.01	701.5	718.3	771.0	797.0	791.0	5	691.0	815.3
**Temp + Salinity**	4	678.0	37	<0.01	702.2	717.9	767.8	797.4	787.3	3	692.1	812.4
**Temp + Salinity + Mass**	5	678.8	37.8	<0.01	703.2	717.9	769.7	796.2	789.2	4	693.4	814.1
Salinity*Mass + Temp	6	680.4	39.4	<0.01	704.9	719.2	770.1	797.8	791.1	5	695.4	816.1
Mass*Temp	5	744.0	103	<0.01	773.0	782.7	811.5	849.8	829.7	4	743.2	830.4
**Mass + Temp**	4	750.9	109.9	<0.01	779.2	784.6	812.7	851.6	829.1	3	749.2	830.6
**Temperature**	3	750.9	109.9	<0.01	778.7	785.7	810.8	849.9	827.2	2	749.2	829.3
**Salinity**	3	904.5	263.5	<0.01	923.4	908.5	976.8	994.0	964.7	2	978.6	906.7
**Mass + Salinity**	4	905.5	264.5	<0.01	920.0	906.1	975.7	996.0	965.1	3	980.1	907.6
Mass*Salinity	5	906.8	265.8	<0.01	919.2	906.3	977.2	997.6	966.6	4	981.5	909.0
**Mass**	3	911.1	270.1	<0.01	931.4	920.4	980.0	1012.1	972.5	2	994.5	912.2

The convention for testing all individual effects and their interactions is used such that for example mass*temperature*salinity indicates that the model estimated the effects of mass, temperature and salinity each separately in addition to the pairwise interaction between each variable and the three-way interaction between all variables. Additive effects with no interactions between variables are indicated with a ‘+’ in the model formula. The ΔAIC and Akaike weights (*w*_*i*_AIC) are given for the Weibull distribution models only as this was consistently the distribution with the best fit. Number of parameters were the same for all models except the Rayleigh and Exponential models and are separated as such.

**Fig 5:**
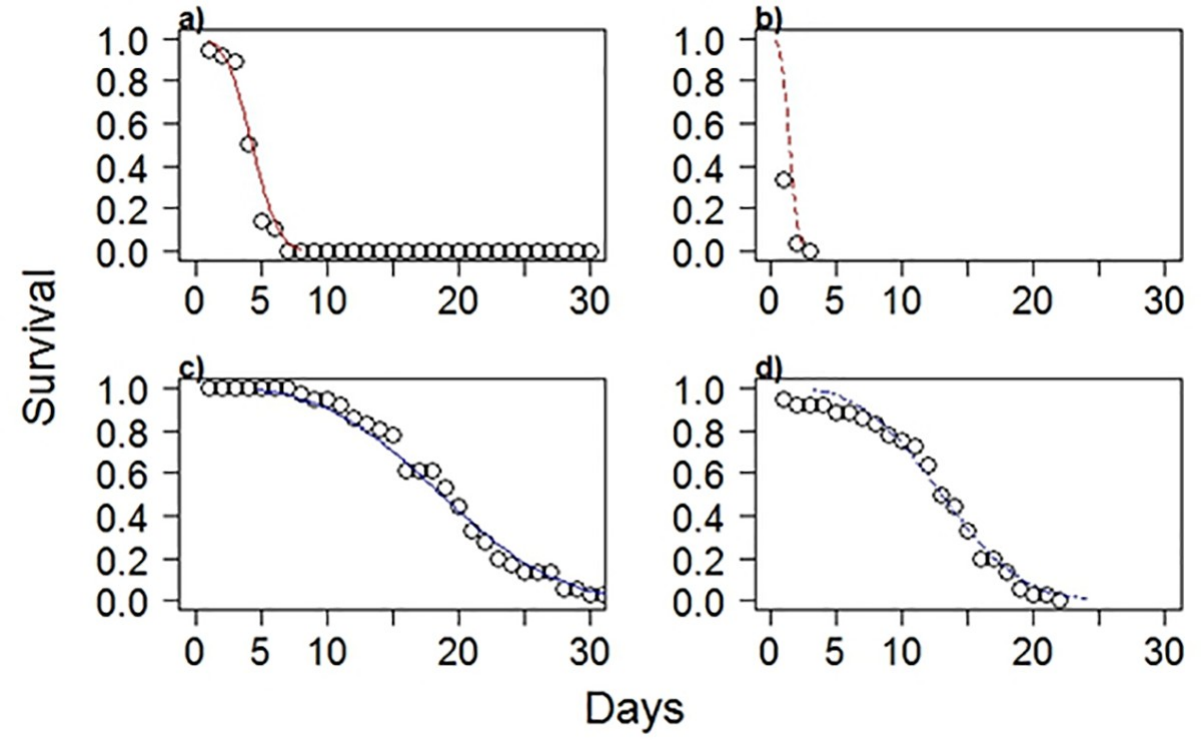
Predicted survival functions of YOY C. *striata* formed from the accelerated failure model with a Weibull distribution (lines) compared to observed survivorship (circles) for **a**) 3°C and 15 PSU, **b**) 3°C and 35 PSU, **c**) 5°C and 15 PSU, **d**) 5°C and 35 PSU.

The survivorship function for the selected model is expressed as function of temperature (*T*), salinity (*S*) and their interaction:

$$S(t,\;\lambda )=\exp\{{-t}^{\lambda }*\exp\left[(-\lambda )(1.078+0.448(T)-0.112(S)+0.018(T*S\right))]\}$$ (1)

where t is winter duration and λ is the shape parameter (Table 3). Predicted values using the Weibull model fit well to the observed data (Fig 5). Predicted survival was highest in the 5°C and 15 PSU treatment followed by the 5°C and salinity 35 (Fig 5). Predicted survivorship was higher than observed survivorship between day 1 and day 2 at 3°C and 35 PSU.

**Table 3:** Test statistics of temperature, salinity and their interaction using a Weibull distribution for YOY *C*. *striata* exposed to two temperatures (3° and 5°C) and two salinities (15 and 35).

Parameter	df	Estimate	SE	Lower 95% CL	Upper 95% CL	Wald chi-square	Pr>chi-square
Intercept	1	1.078	0.302	0.485	1.67	3.57	0.0003
Temperature	1	0.448	0.073	0.305	0.591	6.14	< 0.0001
Salinity	1	-0.112	0.011	-0.135	-0.089	-9.75	< 0.0001
Temperature* Salinity	1	0.018	0.002	0.013	0.024	6.82	< 0.0001
Shape	1	0.323	0.067	-1.261	-0.998	-16.81	< 0.0001

## Discussion

By looking at multiple factors simultaneously, we elucidated potential mechanisms for overwintering mortality in *C*. *striata* and developed a quantitative model of overwintering mortality in this species. We identified 6°C as the incipient lethal temperature for *C*. *striata*, the temperature below which temperature-induced death is the primary cause of mortality [2]. Below this temperature, *C*. *striata* cease feeding, experience weight loss and 100% mortality occurs within 32 days, but the time to death is also highly dependent on salinity. Our experimental work at a wide range of temperatures and salinities suggests that disturbance to the central nervous system and/or osmoregulatory failure at temperatures lower than 6°C are the proximate cause of overwintering mortality in this species and potentially other marine temperate fishes while starvation or other mechanisms are more important factors at higher temperatures [1, 2].

At 3°C and 35 PSU, most YOY *C*. *striata* died within two days, and such a fast mortality rate suggests failure of the central nervous system [2]. Reduced activity of Na^+^, K^-^, and ATPase at low temperatures affects all organ systems, but the central nervous system is much more sensitive than other systems because oxygen demand is highest in brain and nerve tissue compared to muscle or liver tissue [6, 52]. Low temperatures reduce synapse transmissions and effectively impede nerve communication to carry out essential bodily functions [53]. Cold-induced damage to the central nervous system has cascading effects to all other organ systems from which the organism cannot recover.

At 5°C, survival of at least two weeks on average, suggests either osmoregulatory failure, starvation or both as the mechanism, but because the response was heavily modulated by salinity, osmoregulatory failure seems the likely proximate mechanism. When temperatures approach a fish’s lower thermal tolerance, active ion pumping, a temperature dependent process, is hindered such that ideal osmolality within cells cannot be maintained resulting in ion concentrations in the blood plasma that eventually cause death [7–9]. Starvation likely occurs after prolonged exposure to temperatures fluctuating around 5–6°C which is supported by the fact that fish in our experiments fed at 6°C, similar to previous studies, but yet still lost weight [14]. Similarly, white perch, *Morone americana*, was found to have osmoregulation difficulties at 2.5°C, but at 4°C starvation was considered the primary driver of mortality [54]. Few studies of overwintering mortality consider the effects of salinity, but in *Morone saxilitis*, suitable overwintering habitat in the Hudson River estuary was dictated more by salinity than temperature [11].

Results from the short cold exposure experiment, while not conclusive, also suggest damage to the central nervous system or osmoregulatory failure as mechanisms of mortality. High mortality occurred even after temperatures increased from 3°C to 5°C. Although mortality was observed in only half of the fish in the short exposure treatment, the majority of this mortality (77%) occurred after the temperature increased above 5°C, suggesting that low temperature can cause irreversible damage to the fish that eventually leads to death even when water temperature increases. This means that if *C*. *striata* experience even a brief cold-snap in the wild, mortality will be relatively high and year-class success potentially low. These results are supported by a previous laboratory experiment that found that mortality of YOY *C*. *striata* increased once temperature went below 6°C and all *C*. *striata* eventually died even as the temperature began to increase from 3°C to 8°C [14].

Our results fill important gaps in previous studies that have examined the effect of temperature and salinity on growth, survival and metabolism in the northern stock of *C*. *striata*. At 32 PSU, the maximum aerobic scope of adult *C*. *striata* occurs at 24.4°C [55] and the optimal temperature for juvenile growth occurs near this temperature (25.6°C at about 23 PSU) [56], but these two studies did not explore the impact of salinity on optimal growth. When the effect of salinity was examined near optimal temperatures for juvenile *C*. *striata* (22–25°C), slightly higher growth occurred at 20–25 PSU and were reduced at extreme high (30 and 34 PSU) and extreme low (10 PSU) salinities [43, 56]. Similarly, we found that growth was higher at 12°C in high salinity treatments, but near lethal temperatures weight loss at 5°C, was greater in the high salinity treatment than the low salinity treatment. Greater weight loss at high salinity is likely for estuarine fishes because osmoregulatory stress is higher at salinities further from the osmolality of the fish, which for estuarine fishes is 8–12 PSU [40, 57]. In the 3°C treatments there was no statistically significant difference in weight loss between salinities, likely because the fish died too quickly for any differences to emerge. Together these studies show that temperature and not salinity is the primary factor controlling growth, but salinity is a masking factor that can alter growth and is particularly influential at thermal limits [1]. The importance of both temperature and salinity on growth has been documented in other temperate marine fishes with higher salinities often better for growth near optimal temperatures [58, 59].

Hales and Able [14] observed that mortality of YOY *C*. *striata* exposed to ambient seawater increased sharply around 2–3°C, but all individuals in this study experienced the same salinities that varied from 26–31 PSU. Similar to our results, individuals that were exposed to cold snaps eventually died even as the temperature began to increase to 7–8°C, but it is unclear from this study which of the three potential overwintering mechanisms explain the 100% mortality that occurred over many months. Sullivan and Tomasso [56] estimated that the lower mean lethal thermal limit for juvenile *C*. *striata* was 2.7°C at salinities around 23 PSU. Interestingly, Atwood et al. [40] observed the lower lethal limit to be one degree higher (3.7°C) in experiments with *C*. *striata* from the southern stock and noted that these fish ceased feeding at 10°C, much higher than this or other studies of the northern stock of *C*. *striata* [14]. There are likely physiological differences in the genetically distinct northern and southern stocks of *C*. *striata* that should be investigated to understand range shift dynamics of this species along the US east coast [33].

Contrary to our expectations based on numerous studies documenting size-dependent overwintering mortality rates [16, 60–63], initial mass did not substantially improve models of overwintering mortality. Our finding is supported by Hales and Able [14] who also observed that size was not significant to determining survivorship in overwintering YOY *C*. *striata*. Size-dependent mortality often results in smaller fish dying before larger fish because energy reserves of smaller fish are depleted more quickly [15], but this is not always the case. Lankford and Targett [12] observed that smaller fish lived significantly longer than larger fish in YOY *Micropogonias undulatus*, another temperate fish of the Northwest Atlantic, while another study found that mortality was lowest for intermediate sizes of *Menidia* [64]. YOY *C*. *striata* in this study ranged from 1.5-16g, typical sizes of YOY *C*. *striata* found in temperate estuaries [38, 65]. It is possible that focusing on the relatively narrow range of sizes of the YOY *C*. *striata* that we used in experiments prevented us from better quantifying the effect of size and that if we had examined older age classes a strong effect of size would have emerged. Our results may have differed from other studies because size itself is not the critical factor, but rather is a proxy for energy reserves that prevent overwintering mortality [66]. A series of overwintering experiments with YOY *M*. *saxatilis* also found length was not significant in determining survivorship [67].

Even though we did not find size to be a major factor in predicting overwintering mortality, YOY *C*. *striata* began losing weight below 6°C and as such would likely experience overwintering mortality due to starvation if food availability is so low that they were unable to consume enough food to offset the costs of routine metabolism for a long period of time [68]. Smaller fish have a more diverse range of predators than large fish, are more vulnerable to predators because they are more willing to leave refuge to forage, and may be less efficient at escaping predators [69]. Thus, we would expect to see size-dependent mortality at the northern edge of its range as many other field studies in overwintering fish populations have documented [16, 60–63]. In these controlled laboratory experiments, other factors that can lead to overwintering mortality such as predation and disease were not considered. It is highly likely that overwintering mortality is higher than we estimated from laboratory experiments alone if these two additional factors are considered. In nearly all field studies of overwintering mortality, smaller fish appear to have higher mortality than larger fish [14, 63, 70, 71] suggesting that smaller fish and fish in lower physiological condition exposes juvenile fish to higher rates of both starvation and predation mortality [10].

While overwintering mortality may be underestimated when considering temperature and salinity alone, considering the lethal threshold of these two factors may be a tractable way to incorporate physiological information into population models. Current stock assessments and fish population models of many valuable species do not explicitly account for overwintering mortality at all [10]. However, overwintering mortality is particularly important in temperate systems like Northwest Atlantic estuarine and coastal waters where sea surface temperature can fluctuate by 20°C or more between summer and winter [72, 73]. Therefore, estimates of overwintering mortality are increasingly important for building more informative population models [18] and to develop better management plans that are able to meet fisheries management goals. Identifying the temperature thresholds where overwintering mortality is entirely temperature dependent are potentially less difficult to incorporate into population models because additional information on food availability, fish size and energy reserves are not needed to quantify mortality. The dynamics of overwintering mortality above lethal temperatures may very well be large sources of mortality, but incorporating temperature-induced mortality is a useful first step to incorporate environmental mechanisms into population models. Using the accelerated failure model produced in this study, future studies should examine the relative winter survivorship of YOY *C*. *striata* by using historic winter bottom water temperatures and salinities on the Northeast US continental shelf. This will likely provide insight to recruitment dynamics associated with the severity and duration of winter. Estimated survivorship and eventual recruitment of YOY *C*. *striata* will become increasingly important as the Northeast US continental shelf warms, potentially providing suitable overwintering habitat further north.

## Supporting information

S1 File (DOCX)
