# Supplementary material for: Overwintering survivorship and growth of young-of-the-year black sea bass Centropristis striata
Source: PLoS One. 2020 Aug 24;15(8):e0236705. doi: 10.1371/journal.pone.0236705 (PMC7444820; doi:10.1371/journal.pone.0236705)
Supplement: S1 File — (DOCX) [file pone.0236705.s001.docx]

Supplemental material

Juvenile black sea bass were collected as part of the New York State Department of Environmental Conservation (NYSDEC) Peconic Bay Habitat Data Integration Program. The sampling scheme for this survey uses a grid with fixed starting locations and they then trawl in random directions. Table 2.1 lists the starting locations of stations that are sampled in this survey.

Table 2.1 Location of surveys in the PEconic Bay trawl survey from which black sea bass were collected.

| Station | Starting Latitude | Starting Longitude |
| --- | --- | --- |
| 2 | 41.05817 | -72.4051 |
| 3 | 41.05652 | -72.3968 |
| 4 | 41.0524 | -72.374 |
| 5 | 41.03948 | -72.3853 |
| 6 | 41.04118 | -72.3747 |
| 9 | 41.02502 | -72.4412 |
| 10 | 41.02508 | -72.416 |
| 11 | 41.025 | -72.4075 |
| 12 | 41.02562 | -72.3989 |
| 13 | 41.02437 | -72.3755 |
| 14 | 41.02448 | -72.3591 |
| 15 | 41.0251 | -72.3424 |
| 18 | 41.00843 | -72.4242 |
| 19 | 41.00777 | -72.4086 |
| 20 | 41.00123 | -72.3967 |
| 21 | 41.00805 | -72.3757 |
| 22 | 41.00907 | -72.3584 |
| 23 | 41.00837 | -72.3426 |
| 24 | 41.00855 | -72.3256 |
| 29 | 40.99223 | -72.4255 |
| 29 | 40.99222 | -72.4251 |
| 30 | 40.99128 | -72.4081 |
| 31 | 40.99113 | -72.3919 |
| 32 | 40.99142 | -72.3782 |
| 34 | 40.97493 | -72.5092 |
| 38 | 40.9754 | -72.4424 |
| 40 | 40.97468 | -72.4089 |
| 41 | 40.97467 | -72.3919 |
| 43 | 40.95803 | -72.5257 |
| 44 | 40.9588 | -72.5082 |
| 45 | 40.95783 | -72.4918 |
| 46 | 40.95637 | -72.4747 |
| 48 | 40.95802 | -72.4387 |
| 49 | 40.95825 | -72.4251 |
| 50 | 40.95877 | -72.4086 |
| 51 | 40.94145 | -72.5575 |
| 52 | 40.94112 | -72.542 |
| 53 | 40.94223 | -72.5247 |
| 54 | 40.94153 | -72.5087 |
| 55 | 40.94108 | -72.4919 |
| 56 | 40.94157 | -72.4758 |
| 57 | 40.94523 | -72.4588 |
| 60 | 40.92465 | -72.5911 |
| 61 | 40.9281 | -72.5743 |
| 63 | 40.92482 | -72.5411 |
| 64 | 40.92455 | -72.5246 |
| 65 | 40.92467 | -72.5082 |
| 66 | 40.92457 | -72.4916 |
| 67 | 40.92542 | -72.4746 |
| 68 | 40.92042 | -72.4585 |
| 69 | 40.91167 | -72.591 |
| 70 | 40.91358 | -72.574 |
| 70 | 40.9136 | -72.5736 |
| 71 | 40.9148 | -72.5374 |
| 72 | 40.90823 | -72.5242 |
| 74 | 40.90845 | -72.4924 |
